# 4-({4-[Bis(2-cyano­eth­yl)amino]­phen­yl}diazen­yl)benzene­sulfonamide

**DOI:** 10.1107/S1600536810053158

**Published:** 2010-12-24

**Authors:** Giuliana Gervasio, Domenica Marabello, Federica Bertolotti

**Affiliations:** a Dipartimento di Chimica I,F.M. e Centro CrisDi, University of Turin, Via P. Giuria 7, 10125 Torino, Italy

## Abstract

In the title compound, C_18_H_16_N_6_O_2_S, which belongs to the family of azo dyes, the dihedral angle between the benzene rings is 26.16 (7)°. In the crystal, mol­ecules are joined by N—H⋯N and C—H⋯N hydrogen bonds into double chains parallel to the *a* axis.

## Related literature

For the synthesis and properties of azo dyes, see: Wenker (1935 ▶); Ledoux *et al.* (2000 ▶); Viscardi *et al.* (2002 ▶). For a related structure, see: Sasaki *et al.* (2004 ▶).
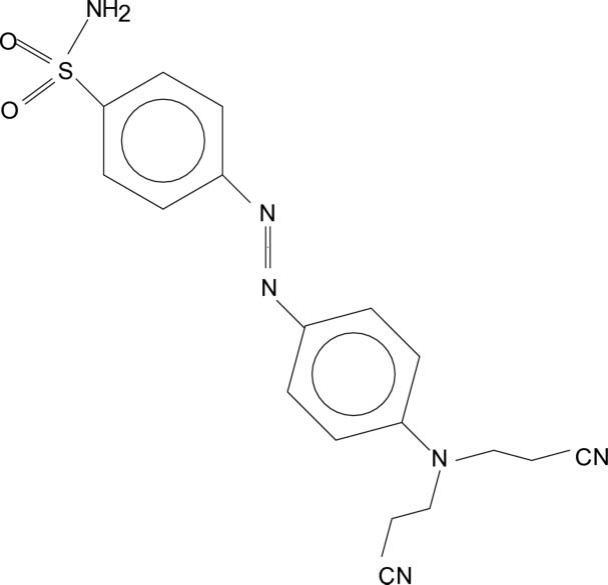


## Experimental

### 

#### Crystal data

C_18_H_18_N_6_O_2_S
*M* *_r_* = 382.45
Triclinic, 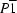

*a* = 7.8093 (16) Å
*b* = 11.035 (2) Å
*c* = 11.776 (3) Å
α = 94.268 (4)°
β = 106.544 (4)°
γ = 104.568 (5)°
*V* = 929.8 (3) Å^3^
*Z* = 2
Mo *K*α radiation
μ = 0.20 mm^−1^
*T* = 295 K
0.30 × 0.23 × 0.03 mm

#### Data collection

Siemens–Bruker APEX diffractometer
Absorption correction: multi-scan (*SORTAV*; Blessing, 1995 ▶) *T*_min_ = 0.93, *T*_max_ = 1.00
9271 measured reflections
4100 independent reflections
1577 reflections with *I* > 2σ(*I*)
*R*_int_ = 0.040
15 standard reflections every 60 min  intensity decay: none

#### Refinement

*R*[*F* ^2^ > 2σ(*F* ^2^)] = 0.050
*wR*(*F* ^2^) = 0.055
*S* = 0.86
4100 reflections
244 parameters
H atoms treated by a mixture of independent and constrained refinement
Δρ_max_ = 0.40 e Å^−3^
Δρ_min_ = −0.48 e Å^−3^

### 

Data collection: *SMART* (Bruker, 2007 ▶); cell refinement: *SAINT* (Bruker, 2007 ▶); data reduction: *SAINT*; program(s) used to solve structure: *SHELXS97* (Sheldrick, 2008 ▶); program(s) used to refine structure: *SHELXL97* (Sheldrick, 2008 ▶); molecular graphics: *XP* in *SHELXTL* (Sheldrick, 2008 ▶); software used to prepare material for publication: *SHELXL97*.

## Supplementary Material

Crystal structure: contains datablocks I, global. DOI: 10.1107/S1600536810053158/rz2541sup1.cif

Structure factors: contains datablocks I. DOI: 10.1107/S1600536810053158/rz2541Isup2.hkl

Additional supplementary materials:  crystallographic information; 3D view; checkCIF report

## Figures and Tables

**Table 1:** Hydrogen-bond geometry (Å, °)

*D*—H⋯*A*	*D*—H	H⋯*A*	*D*⋯*A*	*D*—H⋯*A*
C15—H15*A*⋯N23^i^	0.93	2.52	3.427 (4)	166
N10—H10*B*⋯N27^ii^	0.91 (2)	2.19 (2)	3.084 (2)	165
N10—H10*A*⋯N12^iii^	0.94 (2)	2.19 (2)	3.124 (2)	176

Symmetry codes: (i) 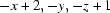; (ii) 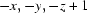; (iii) 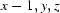.

**Fig. 1.**
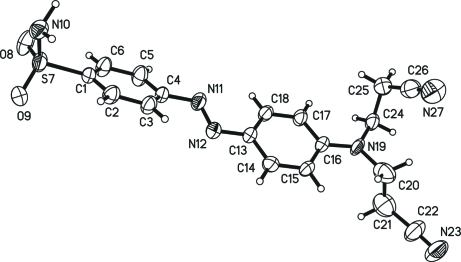
The molecular structure of the title compound showing the atomic numbering and 50% probability displacements ellipsoids.

## supplementary crystallographic information

### Comment

The blood gas (CO_2_ or O_2_) measurement is often required in modern diagnosis and the indicator properties of azo dyestuff have been proved very informative in this field, as proposed in the pioneering work of Wenker (1935). The title compound, 4-diethylcyanoamino-4'-sulfonylamino-azobenzene (Fig. 1), is part of this study and has been synthesized according to a modification of a standard procedure (Ledoux *et al.*, 2000; Viscardi *et al.*, 2002). Bond lengths and angles agree with those of a similar compound reported by Sasaki *et al.* (2004). The two phenyl rings form a dihedral angle of 26.16 (7)°. In the crystal packing, double chains of molecules parallel to the *a* axis are generated by weak C—H···N and N—H···N hydrogen bonds, where N acceptor atoms are the azo (N12) or cyano (N27) atoms (Table 1).

### Experimental

The title compound has been obtained according to Ledoux *et al.* (2000) and Viscardi *et al.* (2002), and prepared by a modification of a standard procedure in analogy to similar compounds previously reported. Crystals suitable for X-ray analysis were obtained by slow evaporation of an ethanol solution.

### Refinement

All H atoms, except those of the NH_2_ group, have been placed in geometrically idealized positions and refined as riding, with C—H = 0.93–0.97 Å and with *U*_iso_(H) = 1.2 *U*_eq_(C). The NH_2_ hydrogen atoms have been located in the final Fourier map and refined freely with *U*_iso_(H) = 1.2 *U*_eq_(N). A small and poorly diffracting crystal has been used in the analysis.

### Crystal data

**Table:**

C_18_H_18_N_6_O_2_S	*Z* = 2
*M**_r_* = 382.45	*F*(000) = 400
Triclinic, *P*1	*D*_x_ = 1.359 Mg m^−^^3^
Hall symbol: -P 1	Mo *K*α radiation, λ = 0.71073 Å
*a* = 7.8093 (16) Å	Cell parameters from 52 reflections
*b* = 11.035 (2) Å	θ = 1.9–20.2°
*c* = 11.776 (3) Å	µ = 0.20 mm^−^^1^
α = 94.268 (4)°	*T* = 295 K
β = 106.544 (4)°	Plate, red
γ = 104.568 (5)°	0.30 × 0.23 × 0.03 mm
*V* = 929.8 (3) Å^3^	

### Data collection

**Table:**

Siemens–Bruker APEX diffractometer	1577 reflections with *I* > 2σ(*I*)
Radiation source: fine-focus sealed tube	*R*_int_ = 0.040
graphite	θ_max_ = 28.4°, θ_min_ = 1.8°
φ scans	*h* = −10→10
Absorption correction: multi-scan (*SORTAV*; Blessing, 1995)	*k* = −14→14
*T*_min_ = 0.93, *T*_max_ = 1.00	*l* = −14→15
9271 measured reflections	15 standard reflections every 60 min
4100 independent reflections	intensity decay: none

### Refinement

**Table:**

Refinement on *F*^2^	Primary atom site location: structure-invariant direct methods
Least-squares matrix: full	Secondary atom site location: difference Fourier map
*R*[*F*^2^ > 2σ(*F*^2^)] = 0.050	Hydrogen site location: inferred from neighbouring sites
*wR*(*F*^2^) = 0.055	H atoms treated by a mixture of independent and constrained refinement
*S* = 0.86	*w* = 1/[σ^2^(*F*_o_^2^)] where *P* = (*F*_o_^2^ + 2*F*_c_^2^)/3
4100 reflections	(Δ/σ)_max_ < 0.001
244 parameters	Δρ_max_ = 0.40 e Å^−^^3^
0 restraints	Δρ_min_ = −0.48 e Å^−^^3^

### Special details

**Table:**

Geometry. All e.s.d.'s (except the e.s.d. in the dihedral angle between two l.s. planes) are estimated using the full covariance matrix. The cell e.s.d.'s are taken into account individually in the estimation of e.s.d.'s in distances, angles and torsion angles; correlations between e.s.d.'s in cell parameters are only used when they are defined by crystal symmetry. An approximate (isotropic) treatment of cell e.s.d.'s is used for estimating e.s.d.'s involving l.s. planes.
Refinement. Refinement of *F*^2^ against ALL reflections. The weighted *R*-factor *wR* and goodness of fit *S* are based on *F*^2^, conventional *R*-factors *R* are based on *F*, with *F* set to zero for negative *F*^2^. The threshold expression of *F*^2^ > σ(*F*^2^) is used only for calculating *R*-factors(gt) *etc*. and is not relevant to the choice of reflections for refinement. *R*-factors based on *F*^2^ are statistically about twice as large as those based on *F*, and *R*- factors based on ALL data will be even larger.

### Fractional atomic coordinates and isotropic or equivalent isotropic displacement parameters (Å^2^)

**Table:**

	*x*	*y*	*z*	*U*_iso_*/*U*_eq_	
C1	−0.4369 (3)	0.3324 (2)	0.1462 (2)	0.0329 (7)	
C2	−0.3729 (3)	0.2269 (2)	0.1422 (2)	0.0385 (7)	
H2A	−0.4475	0.1532	0.0896	0.046*	
C3	−0.1985 (3)	0.2304 (2)	0.2160 (2)	0.0397 (7)	
H3A	−0.1535	0.1603	0.2117	0.048*	
C4	−0.0912 (3)	0.3396 (2)	0.2967 (2)	0.0338 (7)	
C5	−0.1585 (3)	0.4427 (2)	0.3021 (2)	0.0474 (8)	
H5A	−0.0880	0.5146	0.3582	0.057*	
C6	−0.3308 (3)	0.4404 (2)	0.2245 (2)	0.0438 (8)	
H6A	−0.3735	0.5118	0.2258	0.053*	
S7	−0.66133 (10)	0.32583 (7)	0.05031 (7)	0.0441 (2)	
O8	−0.6950 (2)	0.44536 (15)	0.07477 (14)	0.0586 (6)	
O9	−0.6729 (2)	0.27865 (16)	−0.06983 (13)	0.0548 (6)	
N10	−0.8133 (3)	0.2210 (2)	0.0862 (2)	0.0468 (7)	
H10A	−0.817 (3)	0.243 (2)	0.1638 (18)	0.056*	
H10B	−0.799 (3)	0.1427 (19)	0.0698 (19)	0.056*	
N11	0.0845 (3)	0.34976 (19)	0.38119 (16)	0.0416 (6)	
N12	0.1748 (3)	0.28313 (18)	0.34648 (16)	0.0402 (6)	
C13	0.3437 (3)	0.2869 (2)	0.4342 (2)	0.0343 (7)	
C14	0.4404 (3)	0.2039 (2)	0.4088 (2)	0.0406 (8)	
H14A	0.3980	0.1541	0.3336	0.049*	
C15	0.5974 (3)	0.1942 (2)	0.4928 (2)	0.0416 (8)	
H15A	0.6601	0.1382	0.4737	0.050*	
C16	0.6644 (3)	0.2679 (2)	0.6072 (2)	0.0373 (7)	
C17	0.5725 (3)	0.3570 (2)	0.6288 (2)	0.0388 (7)	
H17A	0.6186	0.4110	0.7019	0.047*	
C18	0.4168 (3)	0.3660 (2)	0.5447 (2)	0.0365 (7)	
H18A	0.3586	0.4259	0.5614	0.044*	
N19	0.8150 (3)	0.2551 (2)	0.69548 (18)	0.0471 (7)	
C20	0.9028 (4)	0.1477 (3)	0.6819 (2)	0.0713 (10)	
H20A	0.9460	0.1199	0.7584	0.086*	
H20B	0.8126	0.0760	0.6252	0.086*	
C21	1.0583 (4)	0.1988 (3)	0.6383 (2)	0.0766 (10)	
H21A	1.1448	0.2737	0.6924	0.092*	
H21B	1.0145	0.2216	0.5594	0.092*	
C22	1.1544 (4)	0.0919 (3)	0.6327 (3)	0.0738 (11)	
N23	1.2304 (4)	0.0229 (2)	0.6272 (2)	0.0917 (10)	
C24	0.8777 (3)	0.3258 (2)	0.8154 (2)	0.0445 (8)	
H24A	0.8795	0.4133	0.8098	0.053*	
H24B	1.0046	0.3248	0.8548	0.053*	
C25	0.7577 (3)	0.2751 (2)	0.8932 (2)	0.0538 (8)	
H25A	0.8088	0.3273	0.9717	0.065*	
H25B	0.6327	0.2814	0.8571	0.065*	
C26	0.7480 (4)	0.1423 (3)	0.9075 (3)	0.0547 (9)	
N27	0.7442 (3)	0.0422 (2)	0.9183 (2)	0.0735 (9)	

### Atomic displacement parameters (Å^2^)

**Table:**

	*U*^11^	*U*^22^	*U*^33^	*U*^12^	*U*^13^	*U*^23^
C1	0.0227 (18)	0.0427 (19)	0.0314 (16)	0.0090 (15)	0.0055 (14)	0.0084 (14)
C2	0.0346 (19)	0.0360 (18)	0.0405 (17)	0.0106 (15)	0.0069 (15)	−0.0041 (14)
C3	0.040 (2)	0.041 (2)	0.0427 (17)	0.0215 (16)	0.0118 (15)	0.0065 (15)
C4	0.030 (2)	0.0396 (19)	0.0351 (17)	0.0149 (16)	0.0098 (14)	0.0099 (15)
C5	0.039 (2)	0.045 (2)	0.0466 (18)	0.0112 (16)	−0.0015 (16)	−0.0058 (15)
C6	0.038 (2)	0.041 (2)	0.0496 (18)	0.0204 (16)	0.0025 (16)	−0.0011 (16)
S7	0.0330 (5)	0.0526 (6)	0.0437 (5)	0.0171 (4)	0.0030 (4)	0.0080 (4)
O8	0.0472 (14)	0.0501 (14)	0.0769 (14)	0.0306 (11)	0.0030 (11)	0.0080 (11)
O9	0.0463 (14)	0.0874 (16)	0.0290 (11)	0.0259 (11)	0.0041 (10)	0.0059 (11)
N10	0.0328 (15)	0.0556 (18)	0.0497 (16)	0.0109 (14)	0.0127 (13)	0.0012 (15)
N11	0.0277 (15)	0.0549 (17)	0.0401 (14)	0.0191 (13)	0.0017 (12)	0.0056 (12)
N12	0.0299 (15)	0.0549 (17)	0.0363 (14)	0.0157 (12)	0.0074 (12)	0.0094 (12)
C13	0.0276 (19)	0.0417 (19)	0.0362 (17)	0.0148 (15)	0.0094 (15)	0.0078 (15)
C14	0.039 (2)	0.051 (2)	0.0302 (16)	0.0160 (16)	0.0076 (15)	−0.0014 (15)
C15	0.043 (2)	0.054 (2)	0.0379 (17)	0.0302 (17)	0.0143 (15)	0.0024 (16)
C16	0.0285 (19)	0.049 (2)	0.0380 (18)	0.0181 (15)	0.0089 (15)	0.0108 (15)
C17	0.0333 (19)	0.0476 (19)	0.0336 (17)	0.0197 (15)	0.0023 (14)	−0.0012 (14)
C18	0.0327 (19)	0.0416 (19)	0.0376 (17)	0.0182 (15)	0.0089 (15)	0.0028 (15)
N19	0.0448 (17)	0.0643 (18)	0.0404 (15)	0.0410 (15)	0.0056 (13)	0.0005 (13)
C20	0.049 (2)	0.107 (3)	0.044 (2)	0.001 (2)	0.0126 (18)	0.0098 (19)
C21	0.078 (3)	0.073 (3)	0.064 (2)	0.006 (2)	0.013 (2)	0.016 (2)
C22	0.078 (3)	0.079 (3)	0.080 (2)	0.052 (2)	0.024 (2)	0.008 (2)
N23	0.113 (3)	0.094 (2)	0.121 (2)	0.076 (2)	0.069 (2)	0.040 (2)
C24	0.043 (2)	0.059 (2)	0.0342 (17)	0.0288 (16)	0.0034 (15)	0.0058 (16)
C25	0.064 (2)	0.061 (2)	0.0455 (19)	0.0304 (18)	0.0190 (17)	0.0099 (17)
C26	0.051 (2)	0.061 (2)	0.054 (2)	0.015 (2)	0.0202 (17)	0.005 (2)
N27	0.069 (2)	0.055 (2)	0.095 (2)	0.0141 (18)	0.0277 (17)	0.0099 (18)

### Geometric parameters (Å, °)

**Table:**

C1—C6	1.364 (3)	C15—C16	1.405 (3)
C1—C2	1.381 (3)	C15—H15A	0.9300
C1—S7	1.775 (2)	C16—N19	1.376 (3)
C2—C3	1.381 (3)	C16—C17	1.403 (3)
C2—H2A	0.9300	C17—C18	1.363 (3)
C3—C4	1.387 (3)	C17—H17A	0.9300
C3—H3A	0.9300	C18—H18A	0.9300
C4—C5	1.373 (3)	N19—C24	1.447 (3)
C4—N11	1.419 (3)	N19—C20	1.530 (3)
C5—C6	1.388 (3)	C20—C21	1.452 (3)
C5—H5A	0.9300	C20—H20A	0.9700
C6—H6A	0.9300	C20—H20B	0.9700
S7—O8	1.4326 (15)	C21—C22	1.556 (3)
S7—O9	1.4403 (15)	C21—H21A	0.9700
S7—N10	1.610 (2)	C21—H21B	0.9700
N10—H10A	0.939 (19)	C22—N23	1.085 (3)
N10—H10B	0.913 (19)	C24—C25	1.527 (3)
N11—N12	1.259 (2)	C24—H24A	0.9700
N12—C13	1.414 (3)	C24—H24B	0.9700
C13—C14	1.390 (3)	C25—C26	1.472 (3)
C13—C18	1.393 (3)	C25—H25A	0.9700
C14—C15	1.372 (3)	C25—H25B	0.9700
C14—H14A	0.9300	C26—N27	1.116 (3)
			
C6—C1—C2	120.7 (2)	N19—C16—C17	120.6 (2)
C6—C1—S7	119.93 (19)	N19—C16—C15	121.9 (2)
C2—C1—S7	119.4 (2)	C17—C16—C15	117.5 (2)
C1—C2—C3	120.2 (2)	C18—C17—C16	121.3 (2)
C1—C2—H2A	119.9	C18—C17—H17A	119.4
C3—C2—H2A	119.9	C16—C17—H17A	119.4
C2—C3—C4	119.3 (2)	C17—C18—C13	121.0 (2)
C2—C3—H3A	120.4	C17—C18—H18A	119.5
C4—C3—H3A	120.4	C13—C18—H18A	119.5
C5—C4—C3	120.0 (2)	C16—N19—C24	122.0 (2)
C5—C4—N11	116.5 (2)	C16—N19—C20	122.3 (2)
C3—C4—N11	123.5 (2)	C24—N19—C20	114.63 (19)
C4—C5—C6	120.5 (2)	C21—C20—N19	106.4 (3)
C4—C5—H5A	119.7	C21—C20—H20A	110.4
C6—C5—H5A	119.7	N19—C20—H20A	110.4
C1—C6—C5	119.3 (2)	C21—C20—H20B	110.4
C1—C6—H6A	120.3	N19—C20—H20B	110.4
C5—C6—H6A	120.3	H20A—C20—H20B	108.6
O8—S7—O9	119.73 (10)	C20—C21—C22	106.0 (3)
O8—S7—N10	106.68 (11)	C20—C21—H21A	110.5
O9—S7—N10	106.08 (12)	C22—C21—H21A	110.5
O8—S7—C1	108.04 (11)	C20—C21—H21B	110.5
O9—S7—C1	107.70 (11)	C22—C21—H21B	110.5
N10—S7—C1	108.15 (12)	H21A—C21—H21B	108.7
S7—N10—H10A	112.7 (14)	N23—C22—C21	175.3 (4)
S7—N10—H10B	109.6 (15)	N19—C24—C25	114.2 (2)
H10A—N10—H10B	116 (2)	N19—C24—H24A	108.7
N12—N11—C4	114.1 (2)	C25—C24—H24A	108.7
N11—N12—C13	113.8 (2)	N19—C24—H24B	108.7
C14—C13—C18	118.2 (2)	C25—C24—H24B	108.7
C14—C13—N12	117.5 (2)	H24A—C24—H24B	107.6
C18—C13—N12	124.3 (2)	C26—C25—C24	112.5 (2)
C15—C14—C13	121.2 (2)	C26—C25—H25A	109.1
C15—C14—H14A	119.4	C24—C25—H25A	109.1
C13—C14—H14A	119.4	C26—C25—H25B	109.1
C14—C15—C16	120.6 (2)	C24—C25—H25B	109.1
C14—C15—H15A	119.7	H25A—C25—H25B	107.8
C16—C15—H15A	119.7	N27—C26—C25	178.6 (3)

### Hydrogen-bond geometry (Å, °)

**Table:**

*D*—H···*A*	*D*—H	H···*A*	*D*···*A*	*D*—H···*A*
C15—H15A···N23^i^	0.93	2.52	3.427 (4)	166
N10—H10B···N27^ii^	0.91 (2)	2.19 (2)	3.084 (2)	165
N10—H10A···N12^iii^	0.94 (2)	2.19 (2)	3.124 (2)	176

Symmetry codes: (i) −*x*+2, −*y*, −*z*+1; (ii) −*x*, −*y*, −*z*+1; (iii) *x*−1, *y*, *z*.
